# Death from Starvation

**Published:** 1847-06

**Authors:** 


*Death from Starvation.*—Dr. Leeson said he had been called upon on the previous Monday to make a post-mortem examination in conjunction with Dr. Brady, Professor of Medical Jurisprudence in the College of Physicians, on the bodies of two individuals, a man and woman, who had died suddenly, and, as was reported, from starvation. One of them died on Saturday evening, the other on Sunday morning. Upon external inspection both bodies presented appearances of extreme emaciation. All the features of the female were remarkably contracted, the eyes sunk, nose pinched, and cheeks drawn in, giving her the appearance of a person upwards of sixty years old. The rest of the body was proportionally emaciated; the spaces between the metacarpal bones were perfectly hollowed out; the abdominal parietes when cut were totally devoid of the fat always met with in other subjects, and all the abdominal contents were perfectly bloodless; every organ was completely anæmic.

On cutting into the stomach, its rugæ were observed to be remarkably developed, and there was not a trace of food in it. The whole track of the intestines, though cut into in several places, had not the slightest trace of fœcal matter—containing only a little fœtid mucus. There was some ulceration of the intestinal glands at the lower extremity of the ileum. When the chest was opened, both lungs were emphysematous and perfectly anæmic. The left ventricle of the heart contained half an ounce of remarkably thin fluid blood.

The body of the male presented similar appearances, but the external emaciation was even much greater. The stomach contained a very small quantity of fluid, with a few grains of meal, but no trace of fœculent matter was observed until near the termination of the intestine, and even here its amount did not exceed a teaspoonful. The lungs were in a similarly emphysematous and anæmic condition, and the heart contained a small quantityof fluid blood. The gall-bladder, as usually observed in cases of death by starvation, was remarkably full, and the liver was lobulated, its eminences, however, being of a healthy kind.

In giving an opinion as to the cause of death here, Dr. Leeson remarked that from hearing the facts of the case—that up to the last period of their existence these poor creatures had *something* to live on, it could hardly be distinctly stated that they died of starvation: nor indeed do we find death from absolute starvation to be by any means a common occurrence, taking as an example, for instance, death by starvation, in consequence of several weeks total deprivation of food, as in cases of shipwrecked mariners, &c. In the present instance, however, it appeared that this poor man and woman, with six children, lived in the county Leitrim, and seeing the destruction of the potato crop, made their way up to town, where the whole family were huddled into a miserable apartment, not more than six feet square, with a damp floor and a roof that admitted the rain, a wad of straw that might now have passed for any other material, being its only furniture. Reviewing all the facts of the case then—their destitution previous to coming to town—their bare subsistence while here, having only a sum of 1s. 3d. a day for the support of six children and themselves—life, in fact, as well remarked by Dr. Brady, gradually ebbing away,—the opinion given was that death had arisen not from actual starvation, but from the state of general destitution in which they had so long existed—in want of lodging, fire, clothing, or proper nourishment.

He might here observe, that it is well known the soup obtained by such poor creatures as the above from the soup shops is entirely insufficient for the support of life without some addition of solid material, and this is more particularly true in reference to Paul's parish, where this man and woman lived, the soup there, from the poverty of the parish, being little more than meal and water, which, in the entire absence of solid food in the stomach, passes through the intestinal tract as it is taken in, or gives rise to diarrhœa. The facts of these cases led him to suggest that a certain quantity of solid food might be allowed once or twice a week to the suffering poor in addition to the distribution of soup, and to hope that some notice of the subject might be taken by the public prints.

After finishing the post-mortem examination of the above cases, they proceeded to an adjoining apartment, the area of which was certainly not more than six feet square, and here lay (if he might use the expression) a mere living skeleton, stretched on a portion of straw, the eyes sunken, and their whites suffused, cheeks hollow, nose pinched—in fact, absolutely famishing, unable to move, and just able to call in a voice that reminded him of the voice in cholera for a little water, stating that her mouth was like the burning fire, and it was perfectly parched. The emaciation was so great that her arms were no thicker than candles, and the legs were swollen. She was unable to say when she had tasted food.

In reply to a question, Dr. Leeson stated that no urine was contained in the bladder in either case. Delerium, he believed, too, is only observed in cases of absolute starvation, not in those of gradual starvation, as here.—*Dub. Med. Press.*

